# TKA in the treatment of bilateral dysplasia epiphysealis hemimelica (Trevor’s Disease) of the knee in a 50-year-old man: a case report

**DOI:** 10.1186/s12891-020-3146-3

**Published:** 2020-03-14

**Authors:** Fei Gao, Guo Chen, Ruoyu Wang, Pan Huang, Jing Wang, Weihua Xu

**Affiliations:** 1 Department of Orthopaedics, Union Hospital, Tongji Medical College, Huazhong University of Science and Technology, Wuhan, 430022 China; 2 The First People’s Hospital of Jiangxia District, Wuhan city, China

**Keywords:** Dysplasia Epiphysealis Hemimelica, Trevor’s disease, Total knee Arthroplasty, Radiography

## Abstract

**Background:**

Dysplasia epiphysealis hemimelica (DEH), also known as Trevor’s disease, is a rare skeletal developmental disorder affecting the epiphyses in pediatric patients. DEH is characterized by an asymmetric osteochondral overgrowth arising from either the medial or lateral portion of an epiphysis and usually occurs in the joints of lower limbs, most commonly in the knees and ankles. However, bilateral involvement in an adult is extremely rare, and total knee arthroplasty (TKA) for a patient with DEH has been reported only once before.

**Case presentation:**

Here, we present a case of bilateral DEH of the knees that caused varus deformity and dysfunction of the lower limbs in a 50-year-old man. TKA was performed for treatment, and the patient had satisfactory function with no angular knee deformity and a normal range of motion after 1 year of follow-up.

**Conclusions:**

The patient in this case exhibited its specific clinical and radiological features of late-term DEH and TKA was proved to be an appropriate procedure for treating the severe deformity caused by this rare disease.

## Background

Also known as Trevor’s disease, Dysplasia epiphysealis hemimelica (DEH) is a rare developmental bone dysplasia characterized by excessive growth of cartilage lesions that affect one or more than one long bones, usually in pediatric patients. The condition often occurs in the lower extremities, with the most common sites being the distal femoral epiphyses and tibial epiphyses [1–3]. Although many hypotheses have been proposed [1, 2, 4], the etiology of DEH is still unclear, and there is no evidence of hereditary transmission [5]. The expanding mass on the joint, with or without subsequent valgus or varus deformity or limb length differences, is the main cause of patient visits [2, 6]. Although DEH has been reported in adults, cases are extremely rare, as lesions are usually found in childhood and are subsequently treated by surgical resection. A previously untreated case of DEH involving lesions of bilateral femoral epiphyses in a 50-year-old man is described here. Taking into account the severe varus deformity of the knees and the continuously deteriorating osteoarthritis, the patient accepted our recommendation of total knee arthroplasty (TKA).

## Case presentation

A 50-year-old man presented with pain in both knees with limited motion over 40 years and aggravation for more than a 2-year duration. The patient said that he noticed lumps on the lateral sides of both of his knees since he was 6 years old, and since then, he gradually suffered from a change in gait because of his worsening bowleg and pain in his knees. The limitations in movement progressed throughout his life, but the abnormality was not properly diagnosed and treated until he came to our department with unbearable pain that could not be relieved by painkillers. His medical history showed that he received aortic valve replacement and subsequently took warfarin orally thereafter. The patient’s family medical history was negative.

Physical examination of the patient revealed a waddling gait, with 20 degrees of varus deformity in the knees. The flexion of both knees could approach up to only 110 degrees. The dorsiflexion of both knees could reach up to 5 degrees. A round, well-defined firm mass approximately 3 cm in diameter was palpable proximal to the bilateral patella. Tenderness was negative. The Western Ontario and McMaster Universities Osteoarthritis Index (WOMAC) score was 155, and the visual analogue scale (VAS) score was 6.8.

**Fig. 1:**
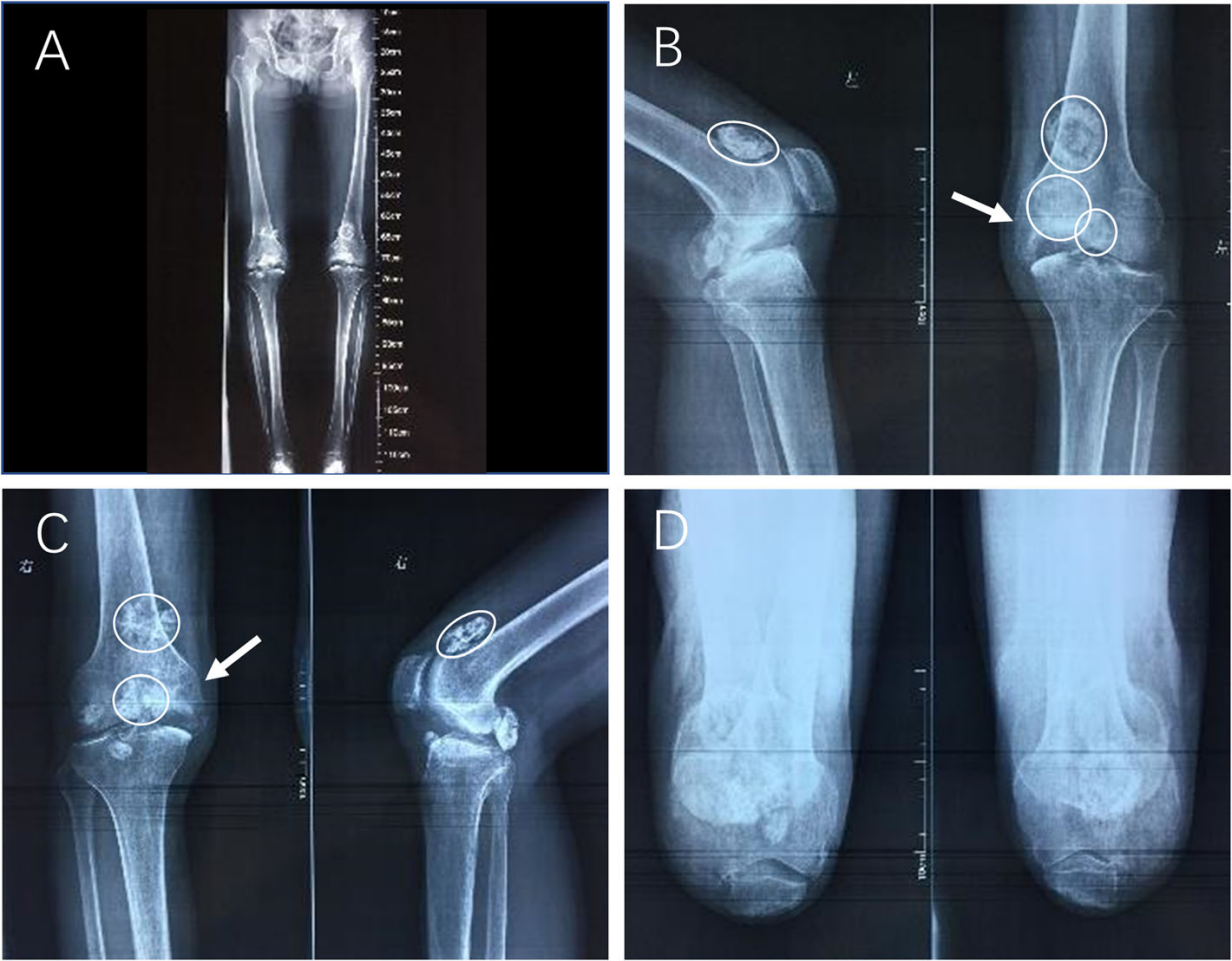
Preoperative plain radiographs. **a** Anteroposterior radiographs of the full lower extremities. **b** Lateral and anteroposterior radiographs of the left knee. The medial femoral condyle is significantly larger than the lateral side. A very large defect as noticed beneath the medial epiphysis of the distal femur (arrow), and several loose bodies were scattered around the medial epiphysis (circle). **c** Anteroposterior radiographs and lateral radiograph of the right knee, which showed similar characteristics as the left knee. **d** Patella axis radiographs of both knees

The anteroposterior and lateral radiographs of the left knee revealed an asymmetric epiphyseal cartilaginous enlargement of the medial epiphysis of the distal femur. A severe deformation of the medial epiphysis was noted due to the presence of a very large hemispherical defect beneath the articular surface. Several loose bodies consisted of cancellous bone with small areas of radiolucency found throughout the medial epiphysis. One of the largest areas of radiolucency was located above the patella. Severe osteoarthritic changes in the lateral compartment were also found. The right knee displayed extremely similar characteristics (Fig. 1).

The clinical manifestations combined with the physical examination and the imaging findings allowed us to arrive at the diagnosis of DEH. Considering the patient’s unbearable pain, severe progressive varus deformity and limited motion, it was determined via a discussion with the patient’s family that continued nonoperative management was no longer appropriate. We could not simply remove the lesion, as it was positioned in a weight-bearing portion of the knee; therefore, a complete corrective surgery was performed.

**Fig. 2:**
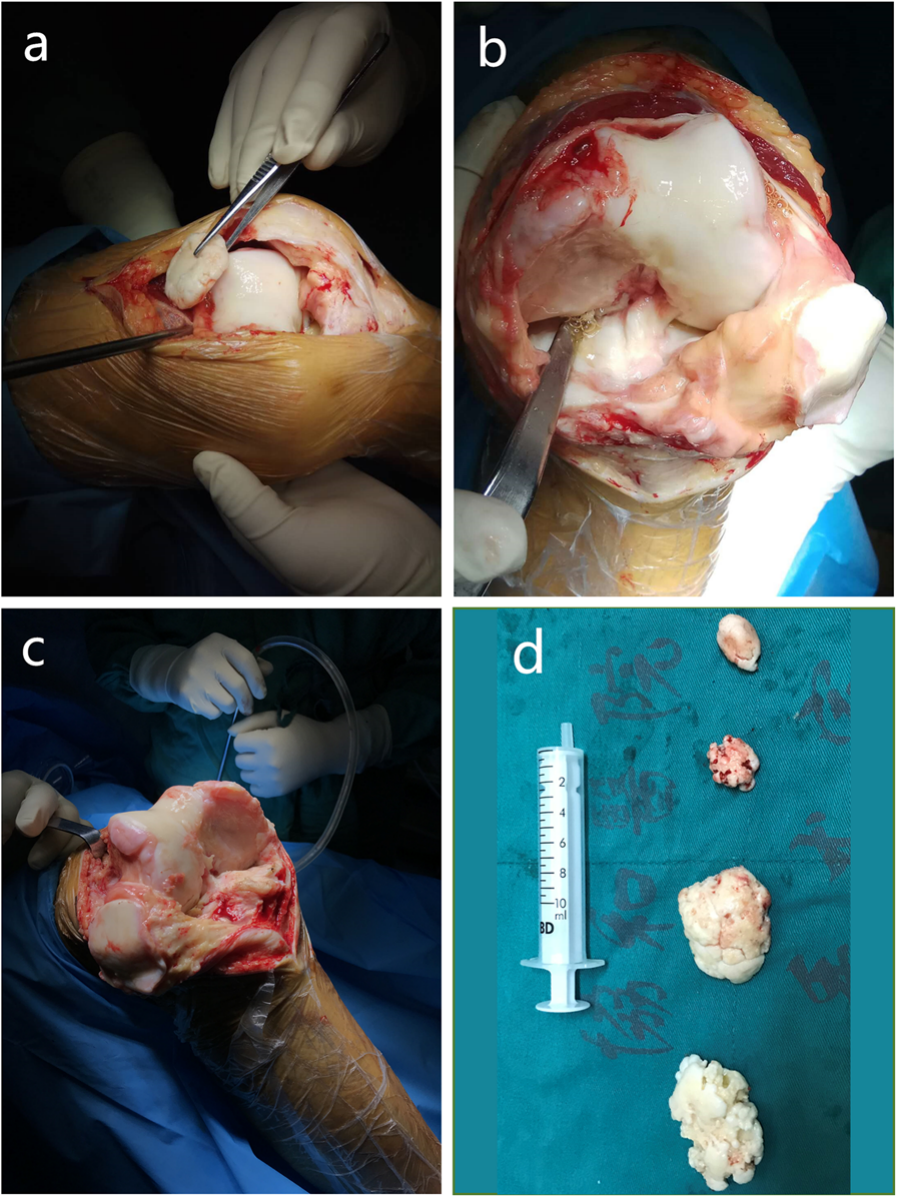
Intraoperative view. **a** The loose bodies were removed. All of the loose bodies were scattered around the medial epiphysis; the largest one was located near the superior patellar bursa and was a round, well-defined bone osteochondral block 3 cm in diameter. **b** The articular surface of the distal medial femur of the left knee was exposed, and a very large defect and a very rough joint surface were noted. **c** The articular surface of the distal medial femur of the right knee was exposed and showed a very similar disorder as that of the left knee. **d** The loose bodies that were removed

**Fig. 3:**
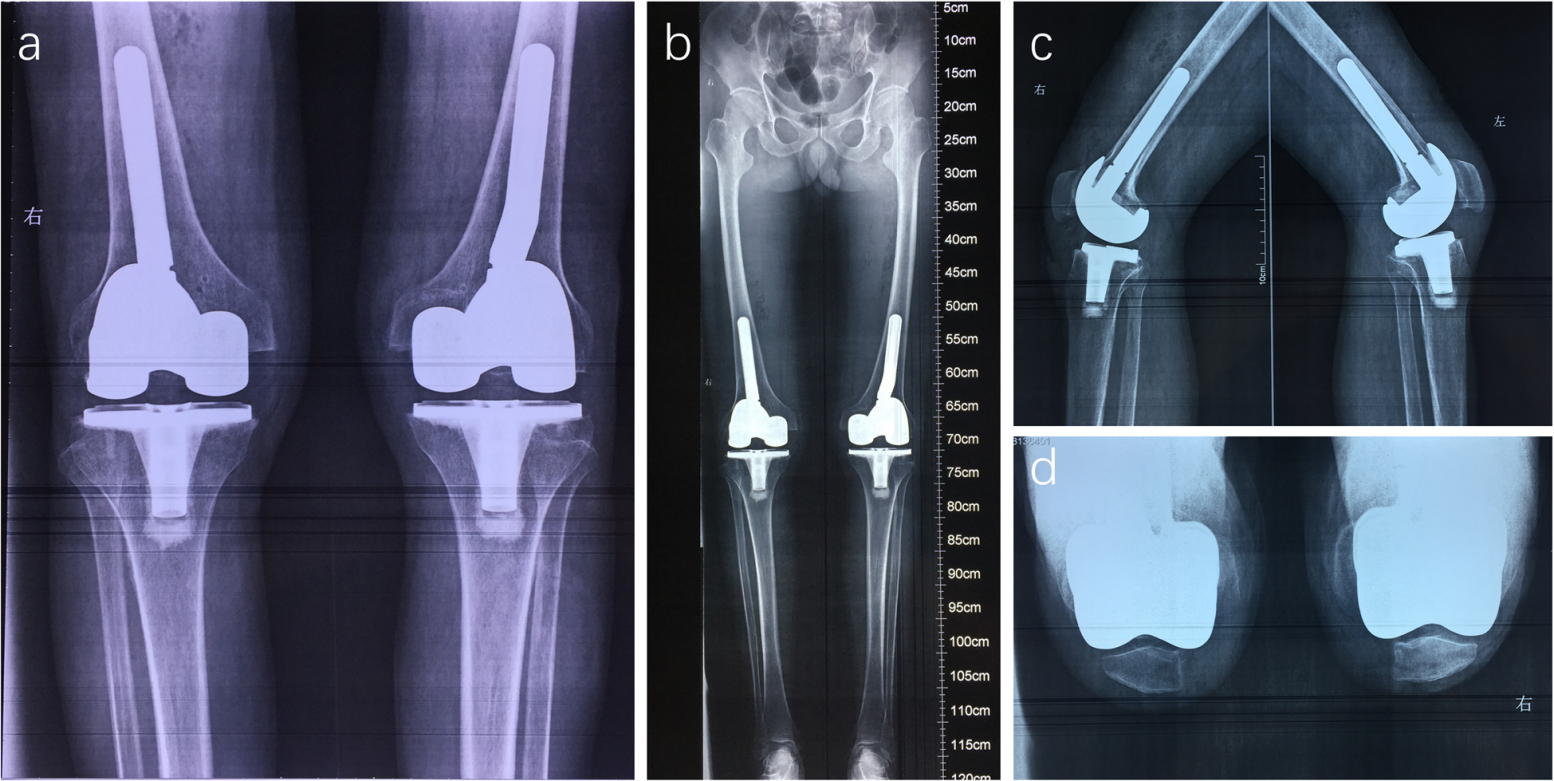
Postoperative plain radiographs. **c** Anteroposterior radiographs of both knees. **b** Anteroposterior radiographs of the full lower extremity. **c** Lateral radiographs of both knees. **d** Patellar axis radiographs of both knees

The patient eventually underwent bilateral TKA (Fig. 2). A benign osteochondroma-like lesion with abnormalities similar to those seen in a sessile osteochondroma was reported by the pathologist. The patient began early-stage functional exercises with the assistance of a walker the day after TKA and returned to work a few weeks later (Fig. 3).

We followed the patient for 2 years after TKA. He exhibited clinical recovery with normal range of motion and was able to walk without lameness or pain. The varus deformity was completely corrected, and the axial alignment of the lower limbs returned to normal. The knee flexion on both sides was close to 70 degrees, and there was no obvious sign of recurrence on the radiographs.

## Discussion and conclusions

DEH is a rare developmental disorder, usually occurring in childhood, in which there are asymmetrical cartilaginous overgrowths of one or more epiphyses of long bones or carpal or tarsal bones [1, 2]. Characteristically, the lesion involved is hemimelic; that is, either the medial or lateral side of the epiphysis is involved [2, 5, 7]. The lesions usually arise from one side of the joint, and thus produce angular and rotational deformities [7]. DEH was first described by Mouchet and Belot in 1926: they defined it a tarsal bone disorder and used the term “tarsomegalie” [3]. The term tarsoepiphysial aclasis was used by Trevor to describe a rare congenital growth disorder of the tarsus and of the epiphysis of the long bones after describing a case in which the lower limbs were affected [1]. Fairbank claimed that the condition should be called “dysplasia epiphysealis hemimelica”, as the condition is characterized by faulty growth of part of one or more epiphyses that is generally confined to the lateral or medial half of a single limb [2]. The etiology of DEH is still uncertain, and there is no evidence to suggest hereditary transmission [5, 7–9]. Malignant transformation has not yet been reported [4, 5, 9–11].

Although the incidence has been reported as 1 in 1,000,000, an increasing number of reports show that the incidence is much higher than that [5, 8, 9]. The age at onset is usually between 2 and 14 years [2, 7, 10]. DEH has been reported in adults, but such cases are extremely rare. Men are affected three times more frequently than women [5, 6, 9].

The condition has a preference for the lower extremities, with up to 73.2% of DEH cases occurring in the lower extremities [9], usually involving the distal femur, proximal tibia, proximal femur, scaphoid and talus [2, 5–7, 9]. The lesion typically involves only one side of the epiphysis, hence the term hemimelica, with rare cases involving the entire epiphysis [9]. The medial side is involved twice as often as the lateral side [11]. The lesion is usually unilateral, and cases that are bilaterally affected, such as our case, are rarely reported.

The formation of a painless, firm bone mass or swelling in the medial or lateral aspect of the affected joint is the most common presenting symptom [2, 8, 10]. Other symptoms include decreased range of motion, atrophy of the muscles that move the affected joints, and dysfunction of the severely involved joints [6–9]. Pain may occur in the ankle [6]. If the protruding mass causes damage to the joint surface, angular deformation of the knee joint (Valgus or Varus) or ankle joint (Valgus or Varus) may occur.

Radiographic findings are usually characteristic and present as an irregular lesion arising from the medial or lateral aspect of the affected epiphysis in the early stages. The calcification center will appear earlier and will eventually undergo multicenter ossification [4, 7, 9, 10]. Although rarely reported, there may be several loose bodies around one side of the epiphysis near the lesion, which we consider to be a characteristic performance in the late stage of the disorder. Secondary single-compartment arthritis is considered a late-term imaging manifestation of the disease. Skeletal survey is recommended once this dysplasia is diagnosed, as there is usually more than one osteophyte involved [8, 11]. Computed tomography can aid in determining the relationship between bone, cartilage and soft tissue [12]. Magnetic resonance (MR) imaging of DEH can accurately show the structural abnormalities of the bone and cartilage in different planes and can reveal secondary disorders in the meniscus, muscles, tendons and ligaments [13].

The diagnosis is primarily based on plain radiographs [9]. DEH should be differentiated from other osteocartilaginous lesions, such as synovial chondromatosis, capsular or para-articular chondroma, tumoral calcinosis, exostosis and, particularly, osteochondroma [4, 7]. DEH and conventional osteochondroma belong to different clinical entities, as indicated by their different locations and clinical presentations. In particular, solitary or multiple osteochondromas arise from the metaphysis or diaphysis, but DEH arises from the epiphysis. Special molecular tests of the genes EXT1 and EXT2 are used for genetic expression analysis to differentiate the entities, as gene expression levels are within normal ranges in DEH, whereas they are lower in osteochondroma. As the radiological appearance is quite specific in most cases, biopsy is considered unnecessary for diagnosis [11]. Nevertheless, biopsy is useful in the early stages of the disease or when the mass is not located in the usual areas.

The treatment of DEH is controversial because of its rarity. Depending on the symptom severity, the extent of the restricted motion and the location of the lesion, the choice of treatment ranges from simple observation to reconstructive surgery of the affected joint [5, 7]. Conservative treatment is recommended for patients without pain or deformity [5, 7, 10]. Even in cases with mild pain and mild limitations of motion, early and active surgery with complete resection of the lesion is widely accepted because the dysplasia is likely to progress and cause severe osteoarthritis and joint deformities [5, 10]. As most of the presenting patients are children, some scholars have warned that multiple intra-articular operations on the affected joint with immature epiphyses might stimulate hyperemia, interfere with bone growth and lead to irreversible joint deformity [6].

Joint replacement is considered an alternative to treating DEH patients with unbearable pain, irreversible malformations, differences in limb length, or failed resection. According to previous reports, only 3 patients diagnosed with DEH had undergone arthroplasty, including 1 shoulder arthroplasty [14], 1 knee arthroplasty [15] and 1 hip arthroplasty [16]. Bilateral involvement is not common for this disorder, as previously mentioned; we consider that there has been no previously reported case treated with bilateral knee arthroplasty. Devine [15] reported an 87-year-old woman who presented with symptoms of this disorder and underwent subsequent knee arthroplasty. However, the absence of typical symptoms, such as painless masses or deformities, as well as atypical imaging findings led us to believe that the diagnosis of this older woman could be further discussed, although the surgery was successfully completed without complications.

DEH, as a skeletal developmental disorder characterized by asymmetric overgrowth of cartilage in the epiphyses in children, is rare, especially bilaterally variations in adults. Our case report presented an adult patient with late-term bilateral DEH in the knees and received TKA which has been shown to be an appropriate method to treat severe irreversible angular deformities. This case report emphasized that DEH should be considered in any case that involves osteochondral tissue and one or more epiphyses and should be diagnosed with the assistance of medical history, physical examinations, imaging and histology.

## Data Availability

The raw data used and/or analyzed during this study are available from the corresponding author on reasonable request.

## Abbreviations

TKA: Total knee arthroplasty
DEH: Dysplasia epiphysealis hemimelica
WOMAC: Western Ontario and McMaster Universities Osteoarthritis Index
VAS: Visual analogue scale
